# Fatal Tick-Borne Encephalitis Virus Infections Caused by Siberian and European Subtypes, Finland, 2015

**DOI:** 10.3201/eid2405.171986

**Published:** 2018-05

**Authors:** Suvi Kuivanen, Teemu Smura, Kirsi Rantanen, Leena Kämppi, Jonas Kantonen, Mia Kero, Anu Jääskeläinen, Anne J. Jääskeläinen, Jussi Sane, Liisa Myllykangas, Anders Paetau, Olli Vapalahti

**Affiliations:** University of Helsinki, Helsinki, Finland (S. Kuivanen, T. Smura, J. Kantonen, M. Kero, A. Jääskeläinen, L. Myllykangas, A. Paetau, O. Vapalahti);; Helsinki University Hospital, Helsinki (K. Rantanen, L. Kämppi, A.J. Jääskeläinen, O. Vapalahti);; National Institute for Health and Welfare, Helsinki (J. Sane)

**Keywords:** Encephalitis, tickborne, tick-borne encephalitis virus, vector-borne infections, flavivirus infections, zoonoses, Finland, viruses, TBEV, meningitis/encephalitis

## Abstract

In most locations except for Russia, tick-borne encephalitis is mainly caused by the European virus subtype. In 2015, fatal infections caused by European and Siberian tick-borne encephalitis virus subtypes in the same *Ixodes ricinus* tick focus in Finland raised concern over further spread of the Siberian subtype among widespread tick species.

The causative agent of tick-borne encephalitis (TBE), tick-borne encephalitis virus (TBEV), is endemic throughout Europe and Asia; ≈10,000 cases are reported annually (*1*). TBEV is an enveloped, positive-sense RNA virus in the family *Flaviviridae*, genus *Flavivirus* (*2*). The westernmost range of the Siberian subtype (TBEV-Sib) extends to Finland and the Baltics, where the European subtype (TBEV-Eur) also circulates. TBEV-Eur is the only subtype found in the rest of Europe (*3*). 

In TBEV-infected patients, neurologic signs appear as the virus passes to the central nervous system; infection is manifested as meningitis, encephalitis, or meningoencephalitis. During 2010–2016, a total of 20 cases of TBE were reported from Kotka archipelago, Finland, a previous TBEV-Sib focus (*4*). We report 2 fatal TBEV infections acquired 1 month apart in patients on Kuutsalo Island, Kotka archipelago, in 2015.

Patient 1 was a previously healthy 36-year-old woman who had visited Kuutsalo 10 days before fever onset. A week later, she experienced sudden-onset headache, left arm numbness, and impaired vision. Head computed tomography results were unremarkable. Two days later, she experienced disorientation and right hemiparesis and was taken to a tertiary care center. Cerebrospinal fluid (CSF) test results showed pleocytosis. Magnetic resonance images indicated pathologically increased signal in cortical sulcus regions (Figure, panel A). Despite receipt of acyclovir, doxycycline, and ceftriaxone, her condition deteriorated rapidly. Head computed tomography showed cerebellar herniation; the patient had dilated pupils and no pain reaction. CSF and serum were positive for TBEV IgM but negative for TBEV RNA; hemagglutination inhibition results showed a low titer (20) of TBEV-specific antibodies in serum. The patient died 2 weeks after fever onset.

**Figure:**
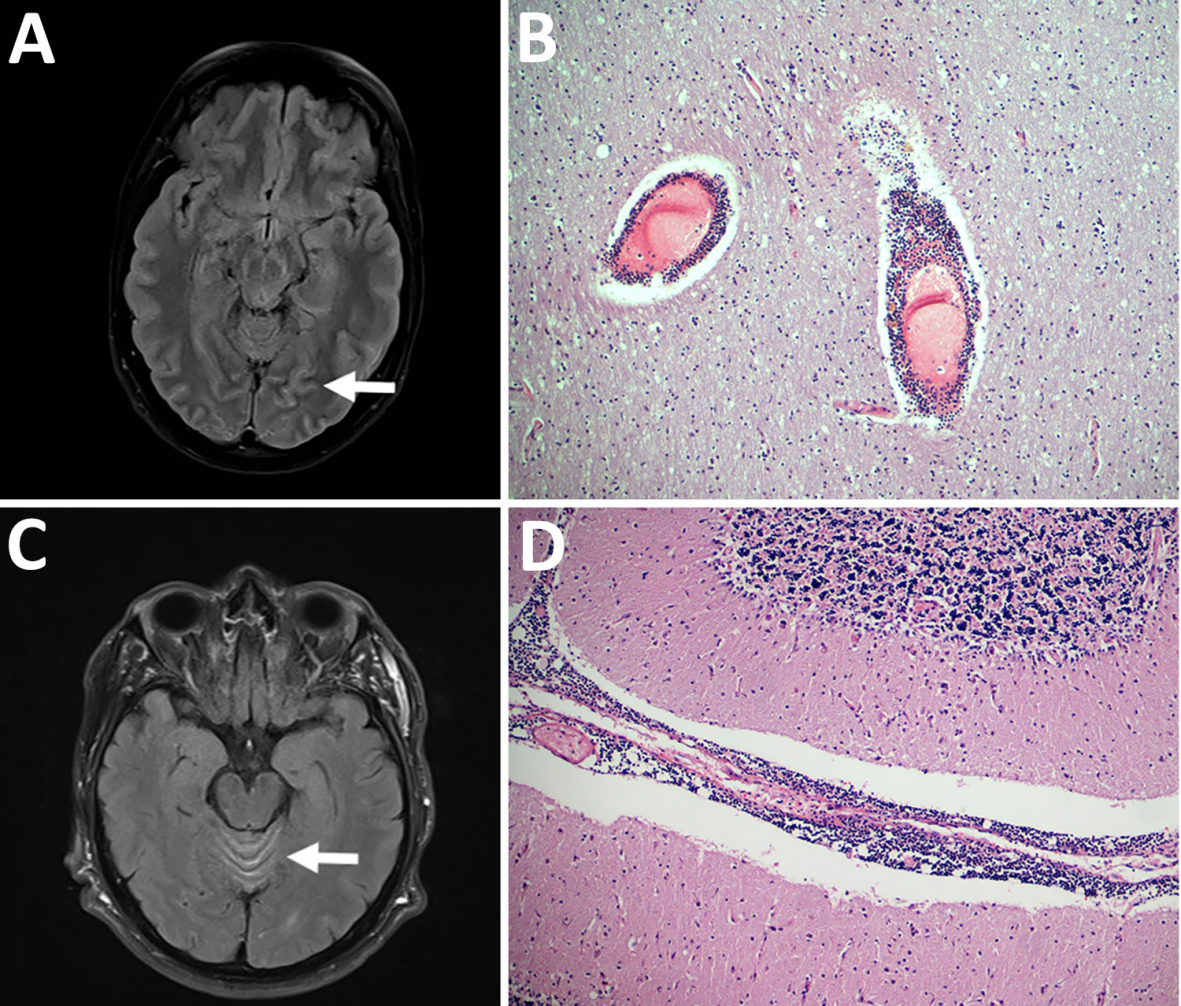
Pathologic and virologic findings for 2 patients with tick-borne encephalitis, Finland, 2015. A) Magnetic resonance images of 36-year-old woman (patient 1) with pathologically increased signal in cortical sulcus regions indicative of viral meningeal process (arrow). B) Hematoxylin and eosin staining of the frontal cortex of patient 1 showed inflammation throughout the central nervous system from the spinal cord to the cortex and cerebellum; original magnification ×100. C) Magnetic resonance image of 66-year-old man (patient 2), showing increased signal in facial nerves, cortical sulci, radicular regions, and cerebellar vermis (arrow). D) Hematoxylin and eosin staining showed microscopically abundant perivascular lymphocytosis in the cerebellum of patient 2; original magnification ×100.

Gross postmortem examination showed widespread and severe signs of viral encephalitis: meningeal and perivascular inflammation, neuronophagy, microglial nodules, endothelial damage, and severe brain edema. The inflammation was evident from the spinal cord to the cerebellum and cortex (Figure, panel B). TBEV (RNA) was detected in brain and spleen (Technical Appendix Figure 1, panel A).

TBEV was isolated from the cerebellum in SK-N-SH neuroblastoma cells, and the whole genome for TBEV-Sib was obtained. A pool of TBEV-Sib–positive *Ixodes ricinus* ticks collected from the neighboring island in 2011 (*4*) was subjected to viral whole-genome sequencing. This virus and the virus from patient 1 had 3 nt differences resulting in 2 aa mutations, R868K (NS1) and V1452A (NS2B), and clustered together in the Baltic clade of TBEV-Sib (Technical Appendix Figure 2).

Patient 2 was a 66-year-old man with hypertension, diabetes, and chronic lymphatic leukemia. He had frequently been bitten by ticks while at his cottage on Kuutsalo Island. Two weeks before hospitalization, he had persistent fever. By the time he was hospitalized, tetraparesis and urinary retention had developed. Magnetic resonance images showed increased signal in cerebellar vermis, facial nerves, cortical sulci, and radicular regions (Figure, panel C). CSF analysis showed pleocytosis. Serum and CSF were negative for TBEV IgM and RNA. The patient’s condition deteriorated rapidly; tetraplegia developed, and he lost consciousness despite treatment with acyclovir, doxycycline, ceftriaxone, plasmapheresis, and immunoglobulin. One week after hospitalization, his CSF was positive for TBEV IgM but his CSF, serum, and urine were RNA negative. Hypogammaglobulinemia was observed. The patient died 4 weeks after hospitalization.

Postmortem examination showed signs of severe coronary disease, cardiac hypertrophy, atherosclerosis in the aorta, and bronchopneumonia. Examination for neuropathology showed abundant perivascular lymphocytosis continuing to brain parenchyma causing glial reactivity and neuronophagy, altogether demonstrating viral encephalitis prominent in the spinal cord, brain stem, basal ganglia, and cerebellum (Figure, panel D). The brain was positive for TBEV RNA (Technical Appendix Figure 1, panel B). A complete genome for TBEV-Eur was sequenced from the cerebellum (Technical Appendix Figure 2). 

In September 2017, a total of 80 ticks were collected from Kuutsalo Island. One, collected at the cottage of patient 2, was positive for TBEV RNA. The virus was isolated in SK-N-SH cells, and a TBEV-Eur genome was sequenced. This virus and the virus from patient 2 had 6 nt differences, resulting in 1 aa difference (F2995Y).

For both patients, progression of TBE was rapid and aggressive; neither patient had been vaccinated. Patient 1, who was young and previously healthy and who was infected with TBEV-Sib, died of brain herniation. Patient 2, who had predisposing conditions, was infected with TBEV-Eur and died of tetraplegia and subsequent complications.

In Finland, TBEV-Eur has been found atypically in *I. persulcatus* ticks, and TBEV-Sib has been found in *I. ricinus* ticks (*4**,**5*). TBEV-infected *I. ricinus* ticks are typically found in Kotka archipelago. The detection of TBEV-Eur from patient 2 was unexpected in a known TBEV-Sib focus. The high sequence similarities between the viruses from patients and ticks confirm that the infections were acquired from Kotka archipelago. This finding suggests that TBEV-Eur and TBEV-Sib co-circulate in Kotka archipelago in *I. ricinus* ticks and raises concern for further spread of TBEV-Sib in this tick species, which is widespread in Europe. The coexistence of 2 virus subtypes and the potential emergence of more pathogenic variants requires further surveys of TBEV epidemiology and consideration of vaccination guidelines.

Technical Appendix Detection of tick-borne encephalitis virus in the brain of 2 patients; maximum clade credibility tree for tick-borne encephalitis virus.
