# Supplementary material for: Fatal Tick-Borne Encephalitis Virus Infections Caused by Siberian and European Subtypes, Finland, 2015
Source: Emerg Infect Dis. 2018 May;24(5):946–8. doi: 10.3201/eid2405.171986 (PMC5938788; doi:10.3201/eid2405.171986)
Supplement: Technical Appendix — Detection of tick-borne encephalitis virus in the brain of 2 patients; maximum clade credibility tree for tick-borne encephalitis virus. [file 17-1986-Techapp-s1.pdf]

Article DOI: https://doi.org/10.3201/eid2405.171986

Fatal Tick-Borne Encephalitis Virus Infections Caused by Siberian and European Subtypes, Finland, 2015

Technical Appendix

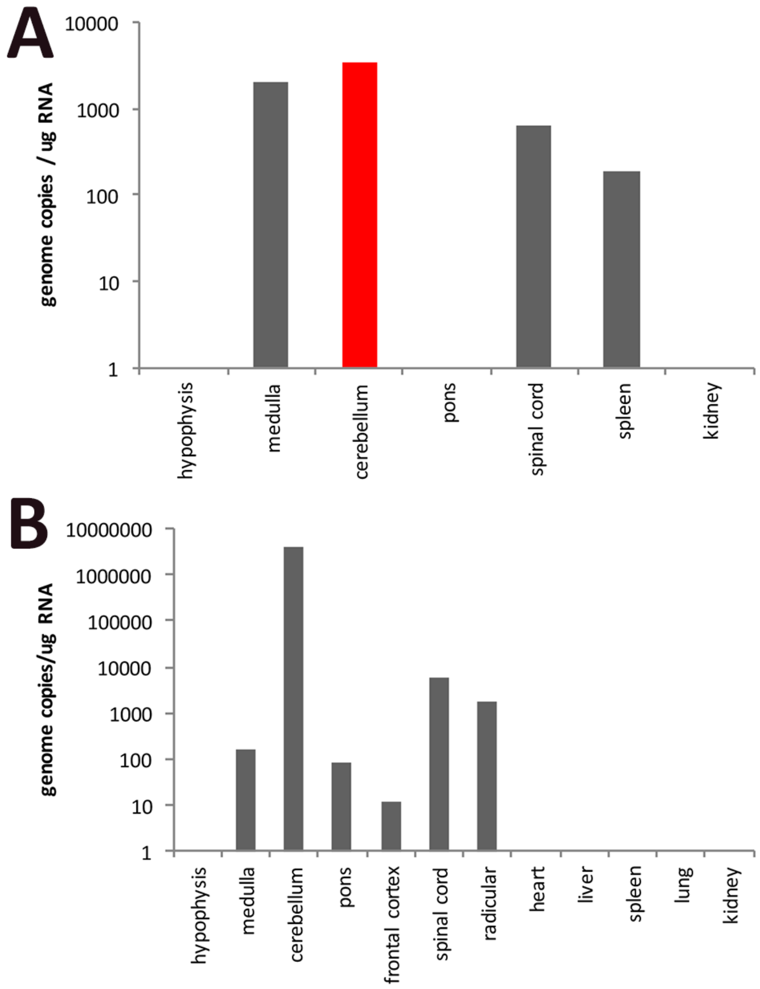

Technical Appendix Figure 1. Tick-borne encephalitis virus (TBEV) RNA detection in brain of patients 1 (A) and 2 (B).

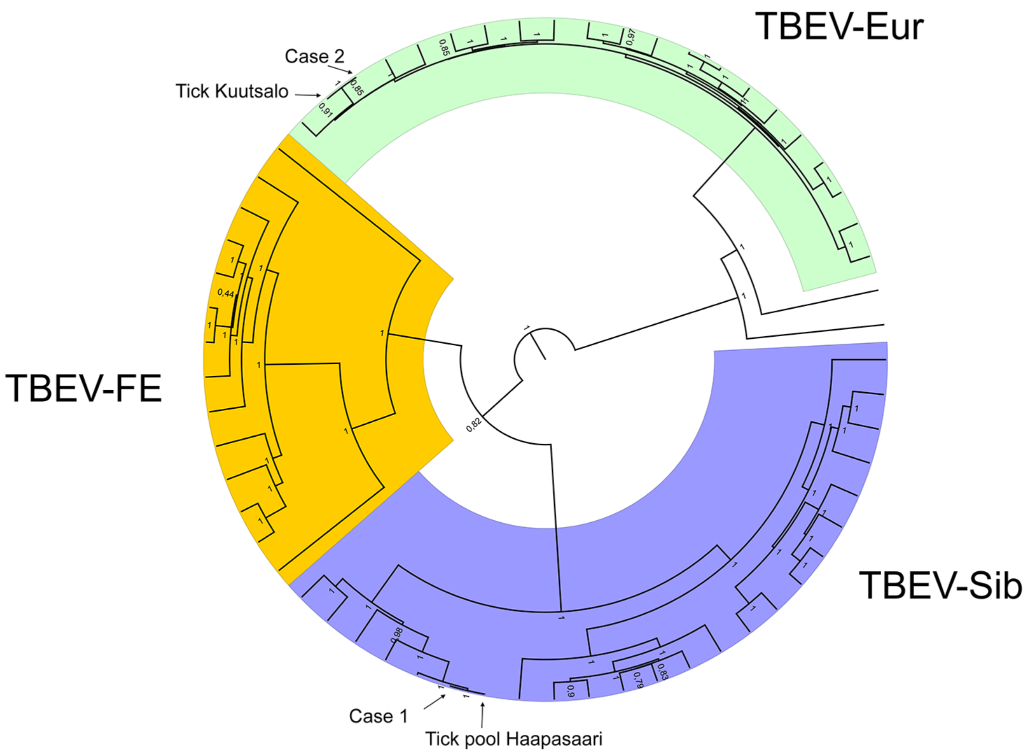

Technical Appendix Figure 2. Maximum clade credibility tree of tick-borne encephalitis virus (TBEV). The phylogenetic trees were constructed from complete coding regions of TBEV-Sib (A) and TBEV-Eur (B) using Bayesian MCMC method with TN93-G-I model of substitution, lognormal relaxed clock model and Bayesian skyline demographic model. Posterior probabilities are shown in each node.
